# Crack Length Effect on the Fracture Behavior of Single-Crystals and Bi-Crystals of Aluminum

**DOI:** 10.3390/nano11112783

**Published:** 2021-10-21

**Authors:** Wilmer Velilla-Díaz, Habib R. Zambrano

**Affiliations:** 1 Departamento de Ingeniería Mecánica, Universidad Autónoma del Caribe, Barranquilla 080020, Colombia; 2 Instituto de Diseño y Métodos Industriales, Universidad Austral de Chile, Valdivia 5110566, Chile; 3 Departamento de Ingeniería Mecánica, Universidad del Norte, Barranquilla 081007, Colombia

**Keywords:** fracture toughness, crack length effect, grain boundary, bi-crystals, single-crystals, molecular dynamics simulations

## Abstract

Molecular dynamics simulations of cracked nanocrystals of aluminum were performed in order to investigate the crack length and grain boundary effects. Atomistic models of single-crystals and bi-crystals were built considering 11 different crack lengths. Novel approaches based on fracture mechanics concepts were proposed to predict the crack length effect on single-crystals and bi-crystals. The results showed that the effect of the grain boundary on the fracture resistance was beneficial increasing the fracture toughness almost four times for bi-crystals.

## 1. Introduction

**Figure 1:**
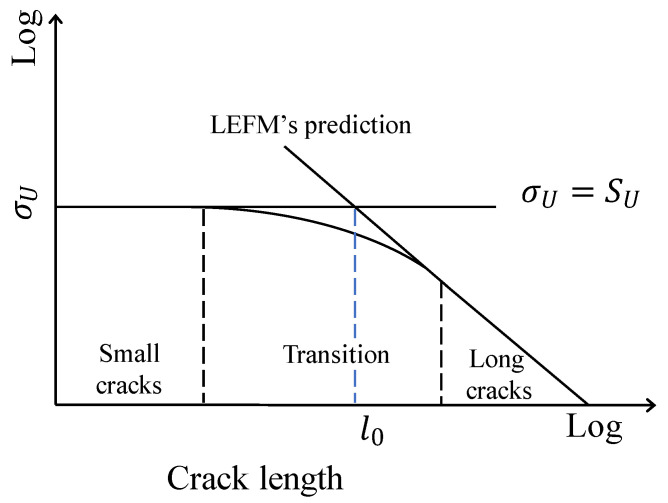
Schematic representation of the crack length effect.

Recently, the development of atomistic models based on molecular dynamics simulations made possible to investigate the mechanical behavior of nanocrystals for a few materials, such as aluminum (Al) [1,2,3,4,5,6,7]. The first analyses were performed to investigate tensile mechanical properties, viz., ultimate tensile strength ($S_{U}$) and young’s module (*E*) [8,9,10,11,12,13]. Later, in order to study the fracture behavior, atomistic simulations of nanocrystals that considered small defects, such as cracks and voids, were developed [14,15,16,17,18,19,20,21]. Based on cracked nanocrystal simulations, it was possible to estimate fracture mechanics properties for some nanocrystal materials. To obtain fracture mechanics properties, such as fracture toughness different fracture mechanics parameter were used to analyze results from molecular dynamics simulations of cracked nanocrystals [22,23,24,25,26,27,28,29]. In addition, some researchers tested the suitability of the fracture mechanics parameters to predict the fracture of nanocrystals [30]. Regarding the fracture behavior of materials at the macroscale, one challenge in fracture mechanics was to obtain an accurate model to describe the effect of the crack length on the fracture of the components. The experiments carried out by different authors (at the macroscale) demonstrated that when a crack was long enough fracture mechanics parameters, such as stress intensity factor (*K*), *J*-integral (*J*), and crack tip opening displacement (CTOD) were suitable to predict the fracture of cracked components, thus the stress at the fracture ($\sigma_{U}$) was defined by the fracture toughness [31] (Figure 1). However, the experiments showed that $\sigma_{U}$ increased rapidly as the initial crack length was smaller, but when the crack was vanishing $\sigma_{U}$ tended to a fixed value which was $S_{U}$ [32] (Figure 1). In order to establish when the crack was long and when it was too small, in other words, when $\sigma_{U}$ was predicted by the fracture toughness and when it was by the $S_{U}$, the characteristic crack length ($l_{0}$) was defined. Crack lengths much larger than $l_{0}$ were long cracks and $\sigma_{U}$ was governed by the fracture toughness, but crack lengths much smaller than $l_{0}$ were small cracks and the $\sigma_{U}$ was defined by $S_{U}$. This crack length effect was extensively investigated at the macroscale by Taylor [32]. A schematic representation of the crack length effect was showed (Figure 1). However, the crack length effect was firstly studied for fatigue failure by Kitagawa et al. [33] and El Haddad et al. [34]. Kitagawa and Takahashi were the first researchers in defining the crack length effect by means of a diagram (Figure 1), but it was applied for fatigue failure analysis of cracked components [33]. In order to analyze the crack length effect in fatigue, El Haddad proposed to modify the stress intensity factor range by adding (to the actual crack length) a constant length that depended on the material properties [34]. Modifying slightly the crack length to calculate the stress intensity factor range, El Haddad obtained an equation that tended to a fixed value (fatigue limit) when the actual crack length was very small, and it led to the usual stress intensity factor range when the crack length was long enough. The El Haddad fatigue model reproduced the Kitagawa-Takahashi diagram [33]. The same concept was adopted and extended to quasi-static monotonic loading failure analysis of components by Taylor in the theory of critical distances [32]. The present investigation was an attempt to extend the same concept into the nanoscale, therefore molecular dynamics simulations of cracked nanocrystals of Al with a wide range of crack lengths were performed in order to study the crack length effect in single-crystals and bi-crystals of Al. The results showed the crack length effect similar to that described above in the cracked nanocrystals. In order to predict the crack length effect on nanocrystals two models were proposed. First, linear elastic fracture mechanics (LEFM) concepts were used to develop an approach to predict $\sigma_{U}$ based on *K*. Second, due to the lack of accuracy of the LEFM model, an elastic–plastic fracture mechanics (EPFM) model was proposed. A novel equation to estimate $l_{0}$ for nanocrystals was formulated. Finally, the effect of the grain boundary on the fracture behavior was also investigated.

## 2. Methodology

### 2.1. Modeling

**Figure 2:**
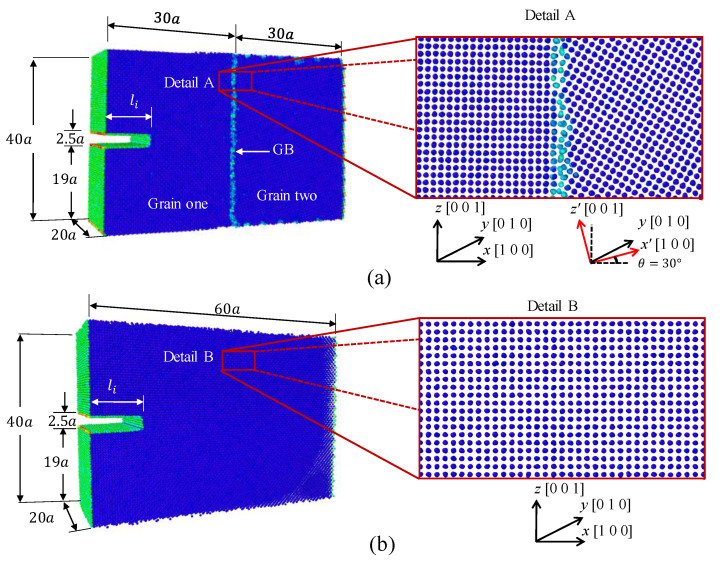
Atomistic system for (**a**) bi-crystal and (**b**) single-crystal Al.

**Figure 3:**
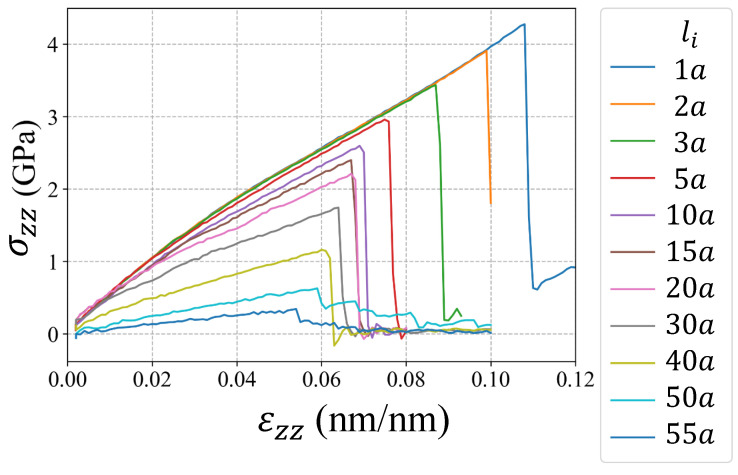
Simulation results of tensile tests for single-crystals Al with different $l_{i}$.

**Figure 4:**
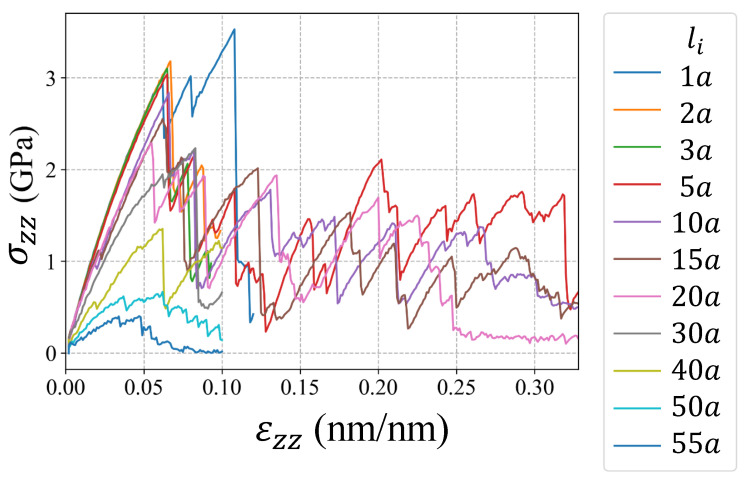
Simulation results of tensile tests for bi-crystals Al with different $l_{i}$.

The molecular dynamics simulations were performed in the code LAMMPS [35]. The atomistic system was first equilibrated using the conjugate gradient method at a pressure of 1.01 bar and at temperature of 300 K using the isobaric–isothermal ensemble (NPT) for 20,000 timesteps of 0.001 ps. The Nose-Hoover barostat and thermostat were implemented to sustain the pressure and temperature. Once the system was equilibrated, loops of deformation-equilibrium were performed until the global stress was close to zero indicating the crystal fracture. Each deformation increment was 0.01% in z-direction with a strain rate of $1\times 10^{-4}$/ps. The length of the atomistic system in the z-direction kept constant during the equilibrium process. The deformation-equilibrium loops were performed using the NPT ensemble applying 10,000 and 20,000 timesteps for deformation and equilibrium, respectively. The embedded atom method potential from Mendelev et al. [36] was implemented, which have been used by other researchers in molecular dynamics simulations of cracked nanocrystals of Al [37,38]. The simulation boxes of the cracked single-crystals and bi-crystals were represented in Figure 2a,b. The dimensions of the simulation boxes were 60a×20a×40a (24.3×8.1×16.2 nm${}^{3}$) for both single-crystal and bi-crystal, where the lattice parameter for Al was a=0.405 nm. To analyze the effect of the grain boundary, a misorientation grain boundary with tilted angle of $30^{\circ }$ was considered in bi-crystal simulations. The crack height was 2.5a for all specimens, and the initial crack lengths were $l_{i}=1a,2a,3a,5a,10a,15a,20a,30a,40a,50a,55a$. The atomistic systems contained approximately 195,000 particles each one. The virial stress tensor was used to compute the global stress [39]. The stress-strain curves obtained from the simulations were presented in Figure 3 and Figure 4 with the corresponding initial crack length $l_{i}$ for single-crystals and bi-crystals, respectively. The volume of the atoms were computed using the Voronoi tessellation in voro++ [40]. DXA [41] was used for dislocation analysis in OVITO [42].

### 2.2. Characteristic Crack Length

The same effective crack length $\left(l_{0}+l_{i}\right)$ proposed by El Haddad [34] to evaluate the crack length effect on the fatigue limit of cracked components was used to estimate the stress intensity factor in mode I ($K_{I}$) for cracked nanocrystals of Al:

(1) $$K_{I}=f\cdot \sigma_{zz}\cdot \sqrt{\pi \cdot (l_{0}+l_{i})}$$

where $\sigma_{zz}$ was assumed to be the global stress (calculated based on the virial stress tensor) in the z-direction and *f* was the geometric factor for edge cracks given by [43]:

(2) $$f=0.265{\left(1-\alpha \right)}^{4}+\frac{0.857+0.265\alpha }{{\left(1-\alpha \right)}^{3/2}}$$

where $\alpha =(l_{0}+l_{i})/60a$. As established in a previous investigation [44], the fracture mechanics parameter *J* was found to be accurate to predict the effect of cracks on single-crystals and bi-crystals. In the same research work different approaches were successfully used for estimating *J* from molecular dynamics simulations. Therefore, in order to use *J*, in this investigation, Equation (1) was rewritten as a function of *J*. To obtain an equivalent expression for Equation (1) in terms of *J*, $K_{I}$ was replaced by $K_{I}=\sqrt{\left(J\cdot E^{\prime }\right)}$ [43], and the following equation was obtained:

(3) $$J=\frac{f^{2}\cdot \sigma_{zz}^{2}\cdot \pi \cdot \left(l_{0}+l_{i}\right)}{E^{\prime }}$$

where $l_{0}$ was possible to determine assuming the limiting conditions for a cracked nanocrystal with a vanishing crack length undergoing $\sigma_{U}$, viz., when $l_{i}\to 0$ and $\sigma_{zz}=\sigma_{U}$, thus $\sigma_{U}=S_{U}$ and $J=J_{C}$ (where $J_{C}$ was the fracture toughness), thus:

(4) $$l_{0}=\frac{J_{C}\cdot E^{\prime }}{f^{2}\cdot S_{U}^{2}\cdot \pi }$$

where $E^{\prime }=E/\left(1-\nu^{2}\right)$ for plane strain and $E^{\prime }=E$ for plane stress [43], and ν was the Poisson’s ratio. Equation (4) showed more explicitly the dependency of $l_{0}$ on the stress state at the crack tip (plane strain or plane stress), which was not evident in the classical formulation proposed by El Haddad [34] for fatigue failure and extended to quasi-static monotonic loading failure by Taylor [32].

### 2.3. Fracture Prediction

**Figure 5:**
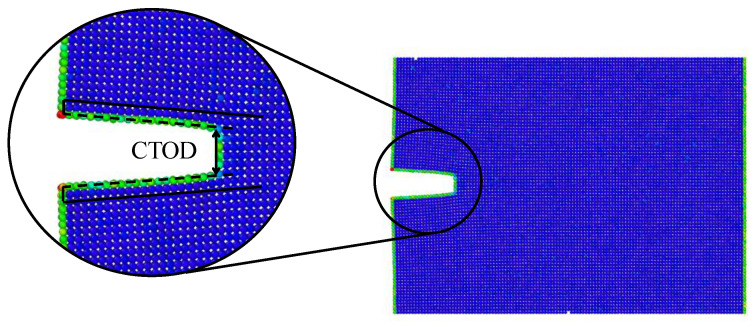
CTOD estimation.

An expression for predicting the crack length effect on $\sigma_{U}$ was obtained by replacing *J* by $J_{C}$ and $\sigma_{zz}$ by ${\bar{\sigma }}_{U}$ in Equation (3), and solving the equation for ${\bar{\sigma }}_{U}$, hence:

(5) $${\bar{\sigma }}_{U}=\sqrt{\frac{J_{C}\cdot E^{\prime }}{f^{2}\cdot \pi \cdot \left(l_{0}+l_{i}\right)}}$$

where ${\bar{\sigma }}_{U}$ was the predicted fracture stress. The predictions of Equation (5) were compared with $\sigma_{U}$ in Section 3 for single-crystals and bi-crystals, where $\sigma_{U}$ was obtained from molecular dynamics simulations by computing the virial stress tensor as mentioned in Section 2.1. However, in order to generate an alternative and more accurate methodology to predict the crack length effect, the EPFM was used by means of the parameter CTOD. Therefore, the CTOD was estimated at the fracture ($CTOD_{U}$) from the simulations and used to calculate an equivalent fracture stress ($\sigma_{Ueq}$). In order to obtain $\sigma_{Ueq}$, *J* was obtained from $CTOD_{U}\left(J_{CTOD}\right)$ as [31]:

(6) $$J_{CTOD}=\frac{\sigma_{U}\cdot CTOD_{U}\cdot \pi }{4}$$

where $CTOD_{U}$ was assumed to be the distance between two selected atoms at the crack tip just before the fracture as proposed in [44,45,46,47]. Figure 5 showed how the CTOD was estimated from the simulations. Finally, $\sigma_{Ueq}$ was obtained assuming $l_{0}=0$, and replacing *J* by $J_{CTOD}$ and $\sigma_{zz}$ by $\sigma_{Ueq}$ in Equation (3), thus:

(7) $$\sigma_{Ueq}=\sqrt{\frac{J_{CTOD}\cdot E^{\prime }}{f^{2}\cdot \pi \cdot l_{i}}}$$

## 3. Results and Discussion

**Figure 6:**
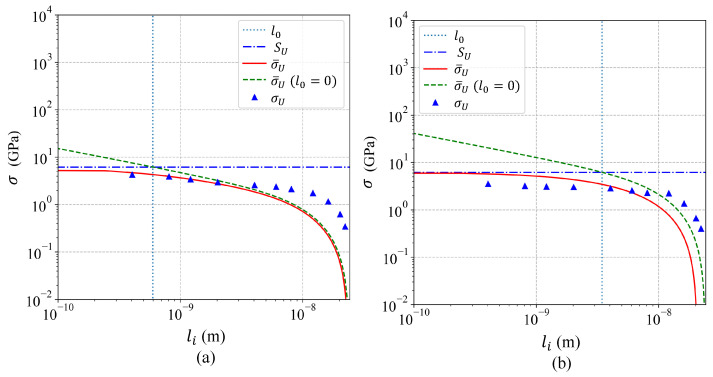
Model based on the LEFM parameter $K_{I}$ and virial stress tensor (**a**) for single-crystals Al and (**b**) for bi-crystals Al.

**Figure 7:**
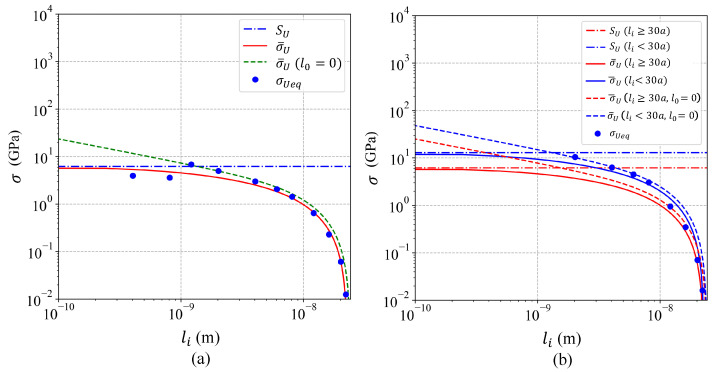
Model based on the EPFM parameter CTOD (**a**) for single-crystals Al and (**b**) for bi-crystals Al.

$J_{CTOD}$ and $\sigma_{Ueq}$ were estimated by means of Equations (6) and (7), respectively, for plane strain, and the results summarized in Table 1. $\sigma_{U}$ and $CTOD_{U}$ were obtained from the simulations and also reported in Table 1. Due to the fact that the fracture toughness was the material property suitable to predict $\sigma_{U}$ for long cracks ($l_{i}>l_{0}$), therefore $J_{C}$ was obtained by fitting Equation (5) to the results obtained from the molecular dynamics simulations for Al crystals with $l_{i}>l_{0}$ (Figure 6a,b). To fit Equation (5) the least squares method was implemented, and the respective $J_{C}$ that minimized the error was reported in Table 2 for single-crystals and bi-crystals. On one hand, Figure 6a,b evidenced that the obtained accuracy was poor, indicating that using only the virial stress tensor to analyze the crack length effect was not suitable. However, Equation (5) was appropriated to reproduce the trend of the results to reach a fixed value ($S_{U}$) when the crack length was vanishing. On the other hand, Figure 7a,b showed that using the EPFM’s parameter CTOD for estimating $\sigma_{Ueq}$ by means of Equations (6) and (7) led to an accurate methodology obtaining consistent estimations for $J_{C}$, where $J_{C}$ was obtained by fitting Equation (5) to the results from long cracks (Figure 7a,b). The $J_{C}$ that minimized the error in Figure 7a,b were presented in Table 3 for single-crystals and bi-crystals. Using this EPFM methodology, the obtained $J_{C}$ coincided with the value reported in [44] for single-crystals. Regarding $J_{C}$ for bi-crystals, the results in Figure 7b indicated two different fracture behaviors. One for crack lengths larger than the selected grain size ($l_{i}\geq 30a$) and other for crack lengths smaller than the grain size ($l_{i}<30a$). Such a behavior was not unexpected, because the grain boundary showed significant effect on the fracture behavior of bi-crystals, while the crack tip was behind the grain boundary ($l_{i}<30a$). However, when the crack tip was beyond the grain boundary ($l_{i}\geq 30a$) the effect on the fracture behavior was irrelevant. In order to investigate the effect of the grain boundary on the fracture behavior, Equation (5) was fitted twice for analyzing bi-crystals. First for bi-crystals with $l_{i}\geq 30a$ and second for $l_{i}<30a$, as shown in Figure 7b, and the results were reported in Table 3. The obtained $S_{U}$ and $J_{C}$ for bi-crystals with $l_{i}\geq 30a$ were almost the same values obtained for single-crystals, therefore the adjusted curves coincided for single-crystals and bi-crystals with $l_{i}\geq 30a$ (Figure 7a,b). The results for bi-crystals showed a substantial drop of 52% and 73% in $S_{U}$ and $J_{C}$, respectively, when the crack length exceeded the grain size indicating that the grain boundary effect was beneficial for the fracture resistance, while the boundary was behind the crack tip. The $S_{U}$ obtained for bi-crystals with $l_{i}<30a$ matched the value reported in [48].

## 4. Conclusions

Based on molecular dynamics simulations, the effect of the crack length was evidenced and investigated on single-crystals and bi-crystals of Al. In addition, the effect of the grain boundary on the fracture behavior was analyzed. The following conclusions were drawn:

- The proposed approach based on the LEFM parameter $K_{I}$ and the virial stress tensor was not appropriate to describe the crack length effect, as shown in Figure 6a,b;
- The proposed approach based on the EPFM parameter CTOD demonstrated to be accurate to predict the crack length effect in single-crystals and bi-crystals, as evidenced in Figure 7a,b;
- The effect of the grain boundary was beneficial increasing the fracture resistance, viz., $S_{U}$ and $J_{C}$, as demonstrated Figure 7b.

## Figures and Tables

**Table 1:** Data used for estimating ${\bar{\sigma }}_{U}$ and $J_{C}$.

Specimen	$l_{i}$ (m)	$\sigma_{U}$ (GPa)	${\mathrm{CTOD}}_{U}$ (m)	$J_{\mathrm{CTOD}}$ (J/m${}^{2}$)	$\sigma_{\mathrm{Ueq}}$ (GPa)
Single-crystal	4.050$\;\times \;10^{-10}$	4.2700	1.349$\;\times \;10^{-9}$	0.3637	3.9434
	8.100$\;\times \;10^{-10}$	3.9098	1.247$\;\times \;10^{-9}$	0.6161	3.5934
	1.215$\;\times \;10^{-9}$	3.4300	1.256$\;\times \;10^{-9}$	3.3830	6.7960
	2.025$\;\times \;10^{-9}$	2.9600	1.370$\;\times \;10^{-9}$	3.1852	4.9659
	4.050$\;\times \;10^{-9}$	2.5600	1.407$\;\times \;10^{-9}$	2.8297	2.9978
	6.075$\;\times \;10^{-9}$	2.3800	1.396$\;\times \;10^{-9}$	2.6093	2.0493
	8.100$\;\times \;10^{-9}$	2.1300	1.428$\;\times \;10^{-9}$	2.3889	1.4288
	1.215$\;\times \;10^{-8}$	1.7436	1.333$\;\times \;10^{-9}$	1.8252	0.6480
	1.620$\;\times \;10^{-8}$	1.1619	1.222$\;\times \;10^{-9}$	1.1147	0.2297
	2.025$\;\times \;10^{-8}$	0.6291	1.720$\;\times \;10^{-9}$	0.8497	0.0609
	2.228$\;\times \;10^{-8}$	0.3432	1.222$\;\times \;10^{-9}$	0.3293	0.0125
Bi-crystal	4.050$\;\times \;10^{-10}$	3.5200	-	-	-
	8.100$\;\times \;10^{-10}$	3.1800	-	-	-
	1.215$\;\times \;10^{-9}$	3.1000	-	-	-
	2.025$\;\times \;10^{-9}$	3.0400	5.869$\;\times \;10^{-9}$	14.0130	10.4158
	4.050$\;\times \;10^{-9}$	2.8400	5.623$\;\times \;10^{-9}$	12.5421	6.3113
	6.075$\;\times \;10^{-9}$	2.5500	6.222$\;\times \;10^{-9}$	12.4602	4.4782
	8.100$\;\times \;10^{-9}$	2.2800	6.063$\;\times \;10^{-9}$	10.8568	3.0459
	1.215$\;\times \;10^{-8}$	2.2318	2.237$\;\times \;10^{-9}$	3.9204	0.9497
	1.620$\;\times \;10^{-8}$	1.3525	2.395$\;\times \;10^{-9}$	2.5440	0.3471
	2.025$\;\times \;10^{-8}$	0.6654	2.174$\;\times \;10^{-9}$	1.1362	0.0704
	2.228$\;\times \;10^{-8}$	0.4012	1.723$\;\times \;10^{-9}$	0.5429	0.0161

**Table 2:** Fracture toughness estimation based on virial stress tensor.

	$l_{0}$ (m)	$J_{C}$ (J/m${}^{2}$)	$S_{U}$ (GPa)	Error
Single-crystal	5.97$\;\times \;10^{-10}$	1.33	6.18	5.6337
Bi-crystal	3.45$\;\times \;10^{-9}$	9.61	6.18	1.6812

**Table 3:** Fracture toughness estimation based on CTOD.

	$l_{0}$ (m)	$J_{C}$ (J/m${}^{2}$)	$S_{U}$ (GPa)	Error
Single-crystal	1.35$\;\times \;10^{-9}$	3.15	6.18	0.2400
Bi-crystal $l_{i}<30a$	1.31$\;\times \;10^{-9}$	13.25	12.92	0.2348
Bi-crystal $l_{i}\geq 30a$	1.53$\;\times \;10^{-9}$	3.60	6.18	0.0095

## Data Availability

Data is contained within the present article.
